# CAM-ICU and ICDSC Agreement in Medical and Surgical ICU Patients Is Influenced by Disease Severity

**DOI:** 10.1371/journal.pone.0051010

**Published:** 2012-11-30

**Authors:** Jorge Alberto de Oliveira Fagundes, Cristiane Damiani Tomasi, Vinicius Rene Giombelli, Sarah Cascaes Alves, Roberta Candal de Macedo, Maria Fernanda Locks Topanotti, Maria de Lourdes Ugioni Bristot, Pedro Emmanuel Alvarenga Americano do Brasil, Márcio Soares, Jorge Salluh, Felipe Dal-Pizzol, Cristiane Ritter

**Affiliations:** 1 Laboratório de Fisiopatologia Experimental and Instituto Nacional de Ciência e Tecnologia Translacional em Medicina, Programa de Pós-Graduação em Ciências da Saúde, Unidade Acadêmica de Ciências da Saúde, Universidade do Extremo Sul Catarinense, Criciúma, Santa Catarina, Brazil; 2 Intensive Care Unit, Hospital São José, Criciúma, Santa Catarina, Brazil; 3 D’or Institute of Research and Education, Rio de Janeiro, Rio de Janeiro, Brazil; 4 Evandro Chagas Clinical Research Institute, Oswaldo Cruz Foundation, Rio de Janeiro, Rio de Janeiro, Brazil; 5 Programa de Pós-Graduação em Oncologia, Instituto Nacional do Câncer, Rio de Janeiro, Rio de Janeiro, Brazil; University of South Florida, United States of America

## Abstract

**Introduction:**

Delirium is a prevalent condition in patients admitted to intensive care units (ICU) associated with worse outcomes. The principal aim of the present study was compare the agreement between two tools for delirium assessment in medical and surgical patients admitted to the ICU.

**Methods:**

Consecutive adult surgical and medical patients admitted to the ICU for more than 24 hours between March 2009 and September 2010 were included. Delirium was evaluated twice a day using the Intensive Care Delirium Screening Checklist (ICDSC) and Confusion Assessment Method adapted to the Intensive Care Unit (CAM-ICU). The kappa (k) and AC1 coefficients were calculated as a measure of agreement between the CAM-ICU and ICDSC.

**Results:**

A total of 595 patients were enrolled in the study. There were 69 (12%) emergency surgical, 207 (35%) elective surgical and 319 (54%) medical patients. Delirium incidence evaluated by the ICDSC, but not by the CAM-ICU, was similar among the three groups. Overall agreement between CAM-ICU and ICDSC was moderate (k = 0.5) to substantial (AC1 = 0.71). In medical patients the agreement between the two instruments was moderate (k = 0.53) to substantial (AC1 = 0.76). The agreement between the two tools in emergency surgical patients was also moderate (k = 0.53) to substantial (AC1 = 0.68). In elective surgical patients the agreement between the two instruments was low (k = 0.42) to substantial (AC1 = 0.74).Agreement rates seemed to be influenced by disease severity. The agreement rate in the general ICU population with APACHE II = <14 was k = 0.57 and AC1 = 0.81, compared to k = 0.44 and AC1 = 0.59, in patients with more severe disease. This was even more different when the need for mechanical ventilation was used as a surrogate of disease severity.

**Conclusions:**

The agreement rates between CAM-ICU and ICDSC may vary between different groups of ICU patients and seems to be affected by disease severity.

## Introduction

Delirium is a prevalent medical condition associated with worse outcomes in patients admitted to intensive care units (ICU) [1], [2]. It is defined by some key features as changes in mental status characterized by a reduced awareness of the environment and a disturbance in attention [3], [4]. Other symptoms as hallucinations, disorientation or temporary memory dysfunction can also occur [5]–[7]. The incidence of delirium in ICU patients ranges from 19% to 87% [8], and postoperative delirium (POD) incidence ranges from 11% to 42% depending on the study population [8], [9]. Such large differences in delirium incidence among the studies can be ascribed to several factors including patients’ characteristics (e.g. type of ICU admission, older age, severity of illness), as well as to the diagnostic tool used to diagnose delirium. The most frequently used instruments to diagnose delirium in the ICU setting are the Confusion Assessment Method for the Intensive Care Unit - CAM-ICU [10] and the Intensive Care Delirium Screening Checklist - ICDSC [11]. The CAM-ICU, adapted from the Confusion Assessment Method, was introduced for the use in mechanically ventilated patients [12]. Originally validated by Ely et al, the CAM-ICU showed a high sensitivity (93%) and specificity (89%) in diagnosing delirium [10]. Lin et al [13] subsequently validated the CAM-ICU in a cohort of mechanically ventilated medical patients and reported similar results. Interestingly, Bergeron and et al validated the ICDSC in ICU patients demonstrating a higher sensitivity as compared to the CAM-ICU (99%) but a lower specificity (64%) [11]. However, the ICDSC is the only tool whose ability to detect subsyndromal delirium has been studied [14].

Direct comparisons between these tools were performed with discordant results [15]–[18]. In addition, to our knowledge, no study has compared these tools specifically in different subgroups as medical and surgical patients. Therefore, the aim of the present study was evaluate the agreement between the CAM-ICU and the ICDSC for delirium diagnosis in different subgroups of ICU patients.

## Methods

### Ethics Statement

The local institutional review board (Research with Humans Ethics Committee of the São José Hospital) approved the present study and written informed consent was obtained from all patients or their legal representatives.

### Study Design, Setting and Patients’ Selection

This was a prospective cohort study performed between March 2009 and September 2010. Consecutive adult (older than 18 years) patients admitted to a 20-bed medical-surgical ICU at a tertiary teaching Hospital for more than 24 hours were included. We excluded readmissions, moribund patients and those with a Richmond Agitation and Sedation Scale (RASS) [19] score equal to-4 or -5 during the entire study period. Clinical data were recorded daily until ICU discharge. Vital status at ICU and hospital discharge were obtained in all patients.

### Delirium Assessment

Delirium was assessed in all patients using both the CAM-ICU and the ICDSC, twice a day (by 08∶00 AM and 02∶00 PM) during their ICU stay. Delirium assessments were performed by investigators fully trained in the use of both scales (CDT, VRG, SCA, TCM, MFLT).

The ICDSC evaluates the level of consciousness, inattention, disorientation, hallucinations, psychomotor activity, speech or mood disturbance, sleep disturbance, and fluctuation of symptoms [11]. According to this instrument, patients were considered to have delirium when at least four of the above mentioned eight items were deviant, and subsydromal delirium was diagnosed in patients with scores between 1 and 3 [11], [17].

According to the CAM-ICU, patients had a diagnosis of delirium when an acute onset of mental status change or a fluctuating course and inattention were accompanied by either disorganized thinking or an altered level of consciousness [10]. The level of consciousness was assessed with the RASS [17], ranging from -5 (unarousable) to +4 (combative).

### Statistical Analyses

Standard descriptive statistics were used to characterize the study population. Continuous variables with normal distribution were presented as mean ± standard deviation and compared by t-Student test or one way ANOVA, as appropriate. Continuous variables with a non-normal distribution were reported as median (25%–75% interquartile range) and compared using Mann-Whitney U test or Kruskal-Wallis test, as appropriate. Categorical variables were presented as absolute numbers (frequency percentages) and analyzed by Chi-square test or Fisher exact test, as appropriate. The diagnostic value of the CAM-ICU and ICDSC were described using 2 X 2 tables. The kappa (k) and AC1 coefficients were calculated, and their correspondent 95% confidence intervals, as a measure of agreement between the CAM-ICU and ICDSC. Agreement was graded as slight (0–0.20), fair (0.21–0.40), as moderate (0.41–0.60), substantial (0.61–0.80) or almost perfect (0.81–1.0). A two-tailed p-value < 0.05 was considered statistically significant. All the analyses were performed with SPSS for Windows, version 17.0, and R-projetc software version 2.15.1.

## Results

**Figure 1:**
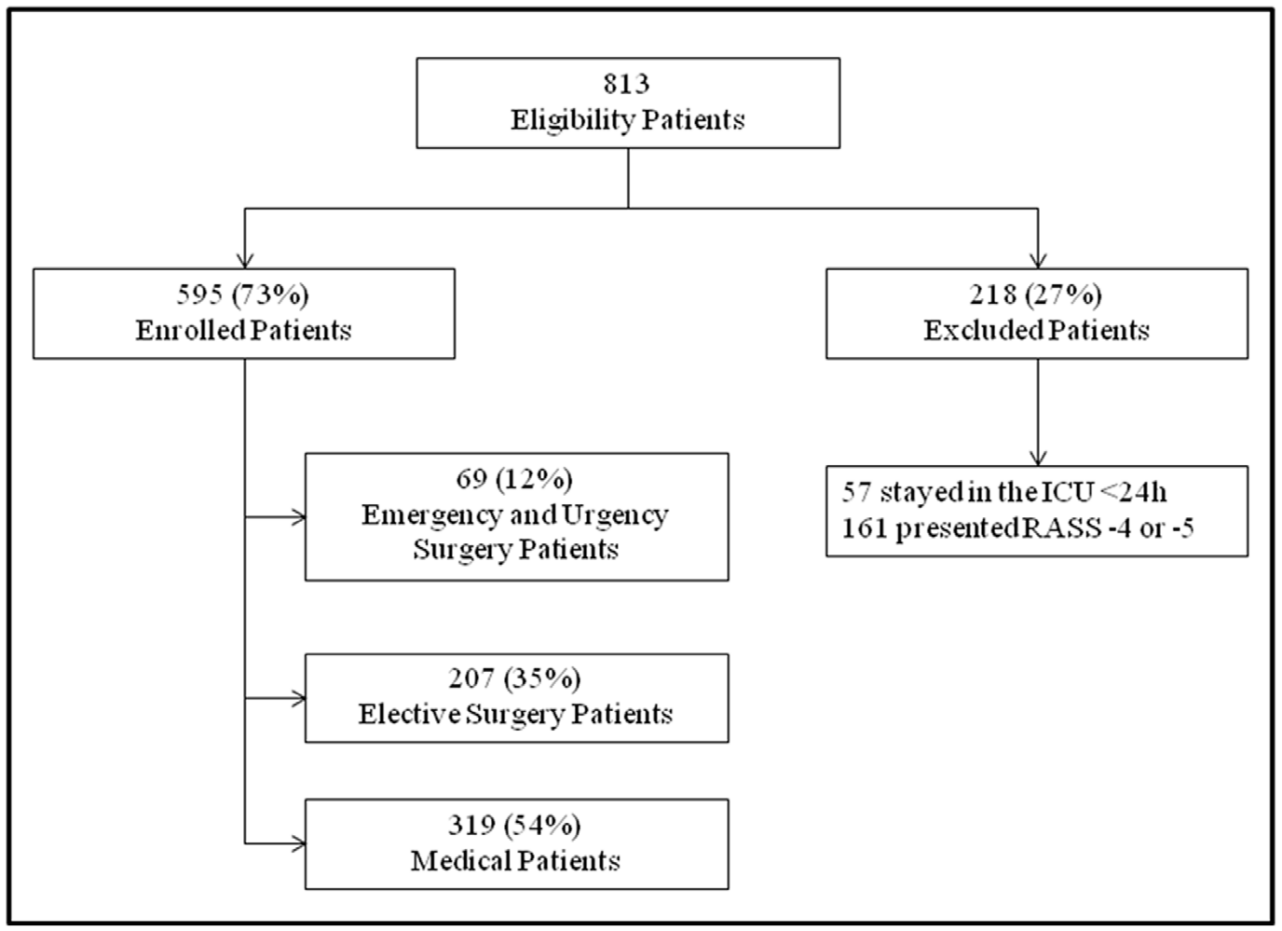
Flowchart of patients in study. ICU = Intensive Care Unit.

During the study period 813 patients were assessed for eligibility, of whom 218 (27%) were excluded (Figure 1). Thus, 595 patients were enrolled into the study, and grouped according to the type of admission into emergency surgical (n = 69, 12%), elective surgical (n = 207, 35%) and medical (n = 319, 54%). The median age and gender distribution were similar among the three groups (Table 1). As expected, the APACHE II (Acute Physiology and Chronic Health disease Classification System II) and SOFA (Sequential Organ Failure Assessment score) scores were significantly higher in emergency surgical and medical patients as compared to elective surgical patients (Table 1). Delirium incidence ranged from 10% to 34.0%, depending on the group of patients and tool used for the diagnosis (Tables 1 and 2). The frequencies of delirium were comparable among the three groups using the ICDSC (Table 1). In contrast, when evaluated by the CAM-ICU, medical patients had a higher incidence as compared to the other groups (Table 1). Overall agreement between CAM-ICU and ICDSC was moderate (k = 0.5) to substantial (AC1 = 0.71) (Table 2). In medical patients, concordant results were found in 258 (81%) patients and the agreement between the two instruments was moderate (k = 0.53) to substantial (AC1 = 0.76) (Table 2). The agreement between the two tools in emergency surgical patients was also moderate (k = 0.53) to substantial (AC1 = 0.68). No patient presented with positive CAM-ICU and negative ICDSC. Concordant results were found in 58 (84%) patients (Table 2). In elective surgical patients, concordant positive results were found in 19 (9%) patients, while concordant negative results were found in 151 (73%) patients. Discordant results were observed in 37 (18%) patients. The agreement between the two instruments was low (k = 0.42) to substantial (AC1 = 0.74) in these patients (Table 2).

**Table 1:** Main patients’ characteristics and type of admission.

		Type of admission			
	All Patients	Emergency Surgery	Medical	Elective Surgery	*p* value
	n = 595	n = 69	n = 319	n = 207	
Age, yrs, median (IQR)	59 (49–69)	57 (49–68)	61 (48–71)	57 (50–67)	0.32
Gender, n (%)					
Male	385 (65)	44 (64)	207 (65)	134 (65)	0.98
APACHE II (points), median (IQR)	14 (9–20)	15 (11–22)	14 (9–22)	13 (8–17) *	< 0.001
SOFA D1 (points), median (IQR)	4 (2–6)	5 (3–7)	5 (2–6)	3 (1–5)*	< 0.001
SOFA D3 (points), median (IQR)	2 (5)	3 (6)	3 (5)	2 (4)*	0.02
Urinary catheter, n (%)	469 (79)	62 (97)	233 (77)#	174 (90)	0.001
Central Venous Catheter, n (%)	400 (67)	56 (88)	175 (59)#	169 (87)	0.001
Enteral nutrition, n (%)	270 (45)	25 (61)	153 (79)#	92 (67)	0.008
Physical restraint, n (%)	81 (14)	14 (20)	47 (15)	20 (10)	0.10
Mechanical ventilation, n (%)	252 (42)	34 (50)	125 (41)	93 (47)	0.24
Sedation, n (%)	184 (31)	26 (38)	110 (36)	48 (23)	0.007
ICDSC positive, n (%)	183 (31)	20 (29)	108 (34)	55 (27)	0.18
CAM-ICU positive, n (%)	96 (16)	9 (13)	67 (21) #	20 (10)	0.001
Lenght of hospital stay (days), mean (SD)	17 (19)	16 (14)	17 (23)	16 (14)	0.80
Hospital mortality, n(%)	108 (18)	11 (16)	77 (24)	20 (9)	0.001

Data are quoted as mean ± SD or number (%).

SD = Standard deviation.

D1 = First day of ICU admission.

D3 = Third day of ICU admission.

* *P*<0.05 from emergency surgery and medical groups.

# *P*<0.05 from emergency and elective surgery.

**Table 2:** Comparison of CAM-ICU and ICDSC for delirium diagnosis.

	*CAM-ICU (n)*							
***ICDSC (n)***								
*All Patients (n = 595)*	Positive	Negative	Total	Kappa [95% CI]			AC1 [95% CI]	
Negative	11(2)	401(67)	412(69)					
Positive	85(14)	98(16)	183(31)					
Total	96(16)	498(84)	595 (100)	0.50 [0.43–0.57]			0.71 [0.66–0.77]	
*Medical Patients (n = 319)*	Positive	Negative	Total					
Negative	10 (3)	201 (63)	211 (66)					
Positive	57 (18)	51 (16)	108(34)					
Total	67 (21)	251 (79)	319 (100)	0.53 [0.32–0.74]			0.76 [0.61–0.90]	
*Emergency Surgical Patients (n = 69)*	Positive	Negative	Total					
Negative	0 (0)	49 (71)	49(71)					
Positive	9 (13)	11 (16)	20 (29)					
Total	9 (13)	60 (87)	69 (100)	0.53 [0.42–0.63]			0.68 [0.60–0.76]	
*Elective Surgical Patients (n = 207)*	Positive	Negative	Total					
Negative	1 (1)	151 (73)	152 (73)					
Positive	19 (9)	36 (17)	55 (27)					
Total	20 (10)	187 (90)	207 (100)	0.42 [0.31–0.54]			0.74 [0.66–0.83]	

Data are expressed as n, %.

Since disease severity could contribute to these differences it was determined the agreement in the subset of patients with APACHE II ≤ 14 and >14 (based on the median of APACHE II score in the sample). The agreement rate in the general ICU population with APACHE II = <14 was k = 0.57 and AC1 = 0.81, compared to k = 0.44 and AC1 = 0.59 in patients with more severe disease. In emergency surgical patients the agreement between the two scales assessed both by kappa and AC1 was similar (Table 3), but it differs in medical patients (k = 0.65 and AC1 = 0.82 compared to k = 0.43 and AC1 = 0.52, respectively). In elective surgical patients, kappa agreement rate was 0.44 and AC1 was 0.80 compared to k = 0.40 and AC1 = 0.65 in patients scored by APACHE II in ≤14 and > 14, respectively. We also attempted to evaluate the effect of severity of illness using the need for mechanical ventilation as a surrogate to identify a more severe group of patients, observing even more different agreements rates (Table 4).

**Table 3:** Comparison of CAM-ICU and ICDSC for delirium diagnosis according to disease severity.

	*CAM-ICU*				
***ICDSC***					
**APACHE II < = 14 points** *All patients (n = 323)*	Positive	Negative	Total	Kappa [95% CI]	AC1 [95% CI]
Positive	38 (11)	41 (13)	79 (24)		
Negative	2 (1)	242 (75)	244 (76)		
Total	40 (12)	283 (88)	323 (100)	0.57 [ 0.46–0.66]	0.81 [0.75–0.87]
*Emergency / Urgency Surgery (n = 39)*	Positive	Negative	Total		
Positive	5 (13)	6 (15)	11 (28)		
Negative	0 (0)	28 (72)	28 (72)		
Total	5 (13)	34 (87)	39 (100)	0.54 [0.26–0.82]	0.77 [0.58–0.96]
*Medical (n = 161)*	Positive	Negative	Total		
Positive	24 (15)	17 (10)	41 (26)		
Negative	2 (1)	118 (73)	120 (74)		
Total	26 (16)	135 (84)	161 (100)	0.65 [0.50–0.79]	0.82 [0.74–0.90]
*Elective Surgery (n = 123)*	Positive	Negative	Total		
Positive	9 (7)	18 (15)	27 (22)		
Negative	0 (0)	96 (78)	96 (78)		
Total	9 (7)	114 (93)	123 (100)	0.44 [0.29–0.58]	0.80 [0.71–0.90]
**APACHE II >14 points** *All patients (n = 272)*	Positive	Negative	Total		
Positive	47 (17)	57 (21)	104 (38)		
Negative	9 (4)	159 (58)	168 (62)		
Total	56 (21)	216 (79)	272 (100)	0.44 [0.33–0.54]	0.59 [0.47–0.68]
*Emergency / Urgency Surgery ( n = 30)*	Positive	Negative	Total		
Positive	4 (13)	5 (17)	9 (30)		
Negative	0	21 (70)	21 (70)		
Total	4 (13)	26 (87)	30 (100)	0.53 [0.21–0.84]	0.75 [0.52–0.97]
*Medical ( n = 158)*	Positive	Negative	Total		
Positive	33 (21)	34 (22)	67 (43)		
Negative	8 (5)	83 (52)	91 (57)		
Total	41 (26)	117 (74)	158 (100)	0.43 [0.28–0.57]	0.52 [0.38–0.65]
*Elective Surgery( n = 84)*	Positive	Negative	Total		
Positive	10 (12)	18 (21)	28 (33)		
Negative	1 (1)	55 (66)	56 (67)		
Total	11(13)	73 (87)	84 (100)	0.40 [0.22–0.58]	0.65 [0.47–0.81]

Data are expressed as n, %.

**Table 4:** Comparison of CAM-ICU and ICDSC for delirium diagnosis according to the need for mechanical ventilation.

	*CAM-ICU*				
***ICDSC***					
**Mechanical Ventilation (no)** *All patients (n = 343)*	Positive	Negative	Total	Kappa [95% CI]	AC1 [95% CI]
Positive	36 (11)	34 (10)	40 (21)		
Negative	5 (1)	268 (78)	273 (79)		
Total	41 (12)	302 (88)	343 (100)	0.57 [0.49–0.69]	0.84 [0.79–0.89]
*Emergency / Urgency Surgery n = 35*	Positive	Negative			
Positive	4 (11)	3 (9)	7 20)		
Negative	0	28 (80)	28 (80)		
Total	4 (11)	31 (89)	35 (100)	0.68 [0.37–0.99]	0.88 [0.75–1.00]
*Medical n = 194*	Positive	Negative			
Positive	25 (13)	18 (9)	43 (22)		
Negative	5 (3)	146 (75)	151 (78)		
Total	30 (16)	164 (84)	194 (100)	0.61 [0.48–0.75]	0.83 [0.76–0.90]
*Elective Surgery n = 114*	Positive	Negative			
Positive	7 (6)	13 (11)	20 (17)		
Negative	0 (0)	94 (83)	94 (83)		
Total	7 (6)	107 (94)	114 (100)	0.47 [0.31–0.63]	0.86 [0.77–0.94]
***ICDSC***					
**Mechanical Ventilation (yes)** *All patients (n = 252)*	Positive	Negative	Total		
Positive	49 (19)	64 (26)	113 (45)		
Negative	6 (2)	133 (53)	139 (55)		
Total	55 (21)	203 (79)	252 (100)	0.41 [0.30–0.52]	0.50 [0.39–0.61]
*Emergency / Urgency Surgery n = 34*	Positive	Negative			
Positive	5 (15)	8 (24)	13 (38)		
Negative	0	21 (61)	21 (62)		
Total	5 (15)	29 (85)	34 (100)	0.44 [0.16–0.71]	0.61 [0.35–0.88]
*Medical n = 125*	Positive	Negative			
Positive	32 (26)	33 (26)	65 (52)		
Negative	5 (4)	55 (44)	60 (48)		
Total	37 (30)	88 (70)	125 (100)	0.40 [0.24–0.56]	0.41 [0.25–0.56]
*Elective Surgery n = 93*	Positive	Negative			
Positive	12 (13)	23 (25)	35 (38)		
Negative	1 (1)	57 (61)	58 (62)		
Total	13 (14)	80 (86)	93	0.37 [0.20–0.54]	0.58 [0.41–0.75]

## Discussion

In the present study, we demonstrated that the agreement rate between CAM-ICU and ICDSC is in general moderate, but varies with the type of ICU admission and severity of disease.

The agreement between scales for delirium diagnosis was the object of a few studies [16]–[18] but, to our knowledge, we provide the first investigation attempting to analyze in separate the agreement between CAM-ICU and ICDSC in medical and surgical patients (elective and emergency surgery) admitted to the ICU and stratified by severity of illness. In a study comparing the agreement between these scales in general ICU patients, kappa’s coefficients ranged from 0.65 to 0.92 [16]. We had previously observed a kappa agreement rate of 0.59 both in a single center study and a multicenter study [17], [18]. Interestingly, even between subsets of surgical patients the agreement rate varies. There are some different characteristics observed in patients that can be related to this variability. Medical and emergency / urgency patients are usually more severely ill at ICU admission as compared to elective surgery patients. In addition, these patients are more prone to use sedation and we can suppose that these differences can interfere in the agreement rate between CAM-ICU and ICDSC. In fact, according to our data it seems that the observed differences in the agreement rates between medical and urgency surgery compared to elective surgery are mainly related to disease severity.

We demonstrated that in the present study population the incidence of delirium did not differ significantly between medical (26%), elective (35%) and emergency surgery (28%) when delirium was evaluated by the ICDSC. In contrast, when evaluated by the CAM-ICU there was a higher delirium incidence in medical patients (20%) when compared to elective surgical patients (10%) and emergency surgical (13%) patients. Several studies had shown that the occurrence of delirium in postoperative patients is common [20]–[22], as it is in the general ICU patients [23]. Patients who were exposed to major surgeries or emergency surgery and developed delirium had more postoperative complications than the patients who never develop delirium [20]–[22]. In addition, medical patients also presented worse outcomes when develop delirium [12]. Nevertheless, delirium is probably under diagnosed [24]. Thus it seems that the low positivity of CAM-ICU in surgical patients indicates that, for this subset of patients, the ICDSC can be a better screening tool. These differences in the performance of the scales also seem to be related to disease severity. In patients presenting with less severe disease delirium positivity was similar in both medical and surgical patients independent on the diagnosis tool that was used. In contrast, in patients presenting with APACHE II score higher than 14 the positivity of CAM-ICU, but not ICDSC, was significantly more frequent in the medical group. The application of CAM-ICU, differently from ICDSC, is more dependent on the interaction between the interviewer and patient, thus is an active diagnosis tool. It is plausible to suggest that as more severely ill, more difficult the interaction between the interviewer and patient (mainly in patients presenting with RASS -3) leading to more difficult tool application. In contrast, ICDSC seems to be more subjective when compared to CAM-ICU, suggesting that its higher positivity is associated a low specificity of delirium diagnosis.

Some limitations of our study must be pointed out. Despite the large sample size this is a single center study. Second, we do not include evaluation of delirium using gold-standard diagnosis by the DSM-IV criteria, thus we can not evaluate sensitivity and specificity of these tool nor ascertain that the differences on CAM-ICU and ICDSC positivity really reflects differences on diagnosis of delirium. This is minimized by the results from a multicenter study demonstrating similar kappa values when comparing CAM-ICU and ICDSC [18]. Third, no statistical analyses were done to compare agreement rates, nor if disease severity is an independent risk factor for agreement of the two delirium assessment tools. We had tried to assess this, but the regression for the concordance that we had performed have, in general, poor discriminative capacity. In addition, kappa analyses are subject to “kappa paradox” which in turn limits the interpretation of agreement through its estimation and a formal (statistic) comparison between two kappa values. We tried to minimize this performing two different agreement analyses, the kappa and the AC1. In addition, from the clinical point of view there is no meaning to determine the variables associated with the agreement between the scales, but we just need to know which tool works better for a determined patient.

### Conclusion

In conclusion, agreement rates between CAM-ICU and ICDSC may vary between different groups of ICU patients and seems to be affected by disease severity.

### Key Messages

- The agreement rate between CAM-ICU and ICDSC is in general moderate, but varies depending on the type of ICU admission and severity of disease.
- Medical and emergency / urgency patients have more severe disease at ICU admission, and they more prone to use sedation and this can interfere in the agreement rate between CAM-ICU and ICDSC.
